# Unexpected colour pattern variation in mimetic frogs: implication for the diversification of warning signals in the genus *Ranitomeya*

**DOI:** 10.1098/rsos.230354

**Published:** 2023-06-07

**Authors:** Ugo Lorioux-Chevalier, Mario Tuanama Valles, Stephanie Gallusser, Ronald Mori Pezo, Mathieu Chouteau

**Affiliations:** ^1^ LEEISA, UAR 3456, Université de Guyane, CNRS, IFREMER, Cayenne, France; ^2^ Instituto de Investigación Biológica de las Cordilleras Orientales, Tarapoto, Peru

**Keywords:** Müllerian mimicry, aposematism, standing variation, evolution, radiation, phenotype

## Abstract

Predation is expected to promote uniformity in the warning coloration of defended prey, but also mimicry convergence between aposematic species. Despite selection constraining both colour-pattern and population divergence, many aposematic animals display numerous geographically structured populations with distinct warning signal. Here, we explore the extent of phenotypic variation of sympatric species of *Ranitomeya* poison frogs and test for theoretical expectations on variation and convergence in mimetic signals. We demonstrate that both warning signal and mimetic convergence are highly variable and are negatively correlated: some localities display high variability and no mimicry while in others the phenotype is fixed and mimicry is perfect. Moreover, variation in warning signals is always present within localities, and in many cases this variation overlaps between populations, such that variation is continuous. Finally, we show that coloration is consistently the least variable element and is likely of greater importance for predator avoidance compared to patterning. We discuss the implications of our results in the context of warning signal diversification and suggest that, like many other locally adapted traits, a combination of standing genetic variation and founding effect might be sufficient to enable divergence in colour pattern.

## 1. Introduction

Phenotypic variation between individuals is the fuel for evolution, as natural selection operates by favouring individuals with traits better suited to the local environment [1]. Because geographical differences in the biotic (e.g. predation, competition, parasitism) and/or abiotic (e.g. salinity, sunshine, temperature) environment can occur, divergent selection can favour distinct phenotypes in different localities [2]. The level of standing genetic variation within a species then determines whether a phenotype that is advantageous in a given environment can be produced, thereby facilitating the colonization of this new environment by this species.

Warning signals displayed in defended prey (i.e. aposematism) are adaptive traits expected to be locally monomorphic [3,4]. The learned avoidance of these signals by predators then results in positive frequency dependent selection: the most common phenotype is favoured. This selection thus promotes the persistence of a single warning signal within a population and the evolutionary convergence of warning signals among both coexisting defended (Müllerian mimicry) [5] and palatable species (Batesian mimicry) [6]. Because the ensuing stabilizing selection is expected to quickly eliminate any individual with a novel (e.g. mutants, recombinant) or exotic warning signal (e.g. migrants), it should effectively limit the amount of variation present in that trait, thus strongly preventing the diversification of warning signals. Yet, while local monomorphism is generally observed within defended species, those species often display a geographical mosaic of distinct aposematic ‘races’ or ecotypes throughout their range [7]. For example, up to 25 distinctive geographical races of the chemically defended butterfly *Heliconus erato* have been documented throughout South America [8,9], while the strawberry poison dart frog, *Oophaga pumilio*, displays up to a dozen geographical morphs in Costa Rica and Panama [10]. The same pattern is observed in *Apheloria* millipedes, with 9 geographical ecotypes, found within a small region of the Appalachian mountains [11], and in the nudibranch *Goniobranchus splendidus*, where 5 distinct geographical morphs have been documented along the eastern coast of Australia [12]. These examples, spanning a broad taxonomic scale, highlight the extensive geographical variation in warning signals.

Both empirical and theoretical studies suggest that this mosaic-like aposematic landscape can be generated by positive frequency dependent selection acting on a randomly distributed diversity of aposematic signals [13]. Such an evolutionary process, once it leads to the fixation of different warning signals in distinct localities, is maintained by its resulting geographical structure of predator that have learned to avoid the local warning signals [4,13–18]. Nevertheless, other selection pressures, that can occur independently or in synergy with predation, may also contribute to spatially structured colour pattern variations. For example, spatial heterogeneity in thermal selection can favour different melanic morphotypes, as in the tiger moth *Arctia plantaginis* [19]. Furthermore, assortative mating based on colour patterns may reinforce barriers to gene flow between races, as in *Heliconius himera* and *H.erato* where males are more likely to approach females displaying their own warning pattern [20]. Finally, geographical variations in aposematic communities can result in warning signal diversification when the prevalence of defended models varies spatially within the range of a mimetic species [13,14]. This phenomenon has been documented in beetles [21], millipedes [11] and dart frogs [22,23].

While the persistence of spatially structured variation in warning pattern has been extensively studied, identifying the mechanisms underlying the initial diversification of warning signals remains challenging. Indeed, decreased phenotypic variability as a result of predators selecting against rare phenotypes should impede the diversification of warning signals. Different hypotheses have therefore been proposed to explain the initial process of this diversification of warning signals, including shifting balance [24–26], whereby genetic drift, novel mutations and recombination, under relaxed predation pressure, generates the variation that enables phenotypic evolution from one adaptive peak to another. Decreased selection pressure associated with increased warning signal variability has been documented in the mimetic poison frog *Ranitomeya imitator* [24], in the dyeing poison frog *Dendrobates tinctorius* [27], and in the tiger moth *A. plantaginis* [28]. Another possible mechanism of diversification is adaptive introgression, whereby gene flow results in the incorporation of a foreign aposematic variant in the species' gene pool, resulting in an important phenotypic leap and potentially providing an immediate local mimetic advantage [29–31]. Another possibility is that predators may associate specific colour pattern elements to defences independently of the rest of the warning signal (such as, for instance, a yellow spot or band; or colour versus pattern) [12,32]. Predators may generalize learned avoidance to all individuals with this pattern element, regardless of the rest of the phenotype, thus allowing phenotypic divergence [7,33]. All these different hypotheses of warning signal diversification nevertheless rely on the maintenance, even transient, of trait variability within populations.

While the expectation for warning signals is the fixation of a single phenotype and mimicry convergence between aposematic species sharing morphological similarities and predators, few studies have documented the extent to which warning signals truly vary within natural populations. Theoretical models of selection provide a conceptual framework to predict how the intensity of selection can shape both warning signal variation and mimetic relationships if these two phenomena are driven by the same selection: an increase in the intensity of stabilizing selection by predators is expected to lead to warning signal uniformity and promote warning signal convergence between unrelated species (mimicry), while a decrease in predation will result in an increase of the warning signal variation (i.e. due to stochastic neutral evolution and/or demographic process counteracting the effect of selection), and mimicry is expected to be absent or imperfect. Therefore, differences in the intensity of predations are expected to generate a negative relationship between the amplitude of variation in warning signals within populations and the degree of mimicry between sympatric species. Yet, if warning signal variation and mimicry convergence are not driven by the same selection, the described negative relationship is not expected to exist.

The Peruvian poison dart frogs, *R. variabilis*, *R. imitator* and species of the *R. fantastica* clade (consisting of the closely related *R. fantastica*, *R. summersi* and *R. benedicta*) provide an ideal biological system to investigate how phenotypic variability in warning signals can be maintained, and how it relates to mimetic relationships. These sympatric species are known for their impressive degree of warning signal ecotypism and for their Müllerian mimetic relationship [34]; indeed, only a few kilometers often separate completely distinct warning forms within a single species (hereafter referred to as ecotypes), and the local warning signal is often shared with the other *Ranitomeya* species living in sympatry. This spatial mosaic of mimetic convergence suggests that the different species of *Ranitomeya* are targeted by the same predator communities, generating locally selected warning phenotypes. Interestingly, previous work on *R. imitator* has located two geographical regions with high phenotypic variability known to be transition zones [24,35]. In one of those regions, polymorphism was facilitated by decreased local selection pressure [24].

In this study, we empirically test the negative relationship between local warning signal variation within a given species and the degree of mimetic convergence between species within natural populations of mimetic *Ranitomeya* poison dart frogs. To test for gradual versus punctuational evolution of colour pattern, we then precisely describe the geographical phenotypic landscape to assess whether warning signals of the different populations (i.e. ecotypes) are characterized either by discrete or continuous phenotypic variation. Continuous phenotypic variation between locally common ecotypes would suggest that standing variation within the metapopulation favours geographical diversification of colour patterns. Finally, we dissected the different components of the warning signals to determine if certain colours or pattern elements are less variable than others, concordant with stronger stabilizing selection on these characteristics than on the rest of the phenotype.

## 2. Material and methods

### 2.1. Sampling

**Figure 1.**
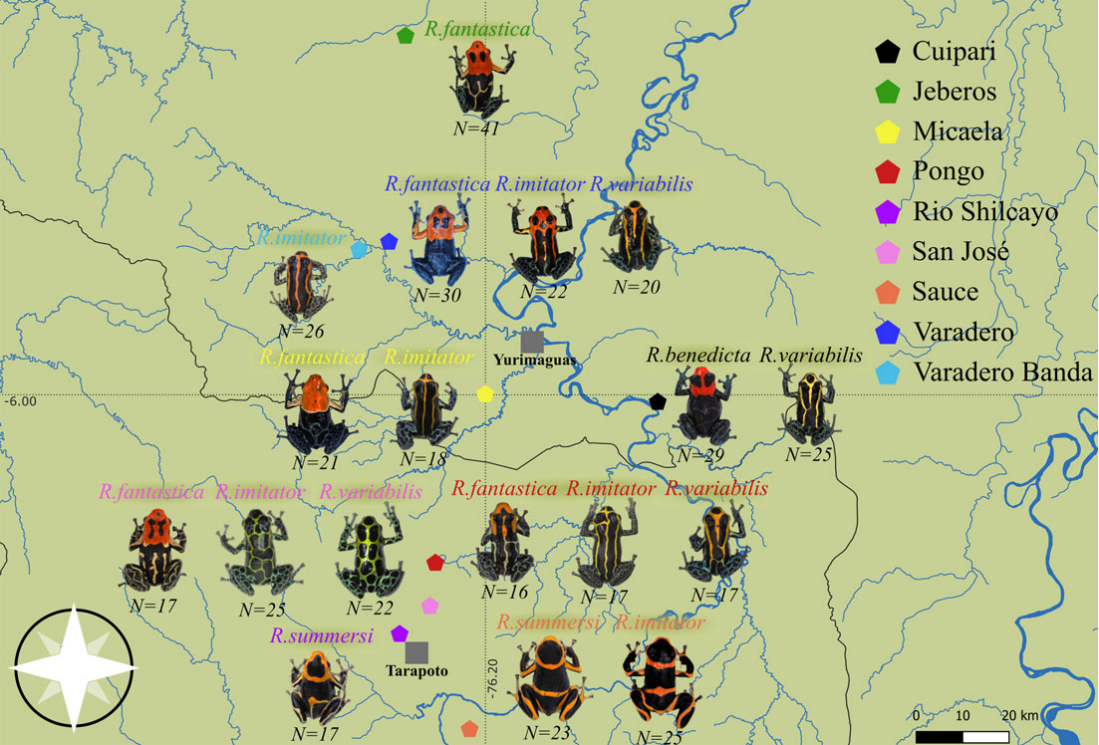
Distribution map of the sampling sites and the associated local ecotypes for all studied species in the region of San Martin and Loreto, Northern Peru.

*Ranitomeya variabilis*, *R. imitator* and the species of the *R. fantastica* clade (*R. fantastica*, *R. summersi* and *R. benedicta*) are amphibians of the *Dendrobatidae* family. Like all species of the genus *Ranitomeya*, these species possess vivid colour patterns and chemical defences (potent skin alkaloids) that deter predators [36]. In the studied region of Northern Peru (San Martin and Loreto department), these species have a different number of ecotypes: 2 mimetic ecotypes have been described for *R. variabilis*, 4 mimetic ecotypes for *R. imitator,* and up to 9 ecotypes for the *R. fantastica* clade [34,35], each characterized by a distinct warning signal. To quantify phenotypic variation within these different ecotypes of *Ranitomeya*, we sampled frogs from 9 localities that differed in the warning signal: Cuipari, Jeberos, Micaela, Pongo, Rio Shilcayo, San-José, Sauce, Varadero and Varadero Banda (electronic supplementary material, table S1, figure 1).

At least 2 species live in sympatry in 7 of the 9 localities. In each locality, all mature frogs encountered were captured and immediately photographed back at the laboratory under standardized conditions using a tripod-supported Olympus EOS 5D Mark III camera with an Olympus 60 mm macro lens. A X-rite ColourChecker Passport Photo 2 was used in every picture to get an accurate correction for luminosity and colours between frogs. Four hundred and eleven *Ranitomeya* frogs of the 3 species (i.e. 195 *R. fantastica* clade, 132 *R. imitator* and 84 *R. variabilis)* were photographed.

### 2.2. Colour and pattern quantification

Quantifying the warning signal is challenging due to the multiple pattern elements and colours that need to be analysed simultaneously to capture the whole visual signal. Here we used a combination of methods to capture the different types of colour and pattern variation. First, frog pictures were standardized for colours using the X-rite colour standard and the X-rite 2019 ColorChecker Camera Calibration V1.1.3 software. We then divided the frog pictures into three body parts: head, dorsum and hind legs, as we generally observe a transition of colours and patterns between these parts. For each of the 3 body parts, we quantified both the colour and the shape of the non-black elements, resulting in a total of 6 phenotypic characteristics.

To quantify the colour of the warning signal, we used the Hue, Saturation, Brightness (HSB) domain [37] using the software Photoshop. Using the magic wand tool in Photoshop CC 2019, we selected the area of interest and extracted the mean HSB value of the body part. The brightness (B) value was then removed from the HSB value as this consists of the luminance present where the frogs were photographed, which fluctuates depending on the amount of light present. Furthermore, although poison frogs are attacked by a variety of predators (snakes, spiders, ants…) [38], birds which are able to recognize colour across Hue and Saturation under diverse conditions of brightness [39] are believed to be the major agent of selection on these frogs' aposematic signal [40,41].

To quantify the pattern, we used three different methods. We first used the R package Patternize [42] to characterize the head pattern. The head pattern was analysed using the pixel-pixel homology comparison of stacked images implemented in Patternize. Prior to using Patternize, 10 landmarks were therefore positioned on all frog pictures using imageJ enabling the precise stacking of the patterning elements (electronic supplementary material, figure S1). To limit the analysis to only the head, the head pattern was manually coloured in red using Gimp, and Patternize was instructed to only extract and analyse this colour. Warning signal pattern elements of the back and the legs range from wide transversal bands to fine reticulation. While the developmental process controlling this patterning is unknown in amphibians, they are reminiscent of the processes of reaction–diffusion patterning [43]. Because this process has a certain randomness, the patterns produced are not adequately analysed by Patternize (i.e. the position of the reticulations of two equally reticulated frogs will always be different) and this results in similarly reticulated frogs being considered as very different. Hindlimbs and dorsal patterns were thus quantified using the ‘adjacent method’ [44]. This method is based on transitions between colour patches, which amounts to describing the amount of reticulation (the alternation of black background and colour pattern). To implement this method for quantifying the dorsal pattern, two parallel transects on each side of the spine, from the neck to the groin on the back, were sampled. Along these transects we counted the number of colour changes, effectively quantifying the degree of reticulation; i.e. from a simple lateral line to a complex network of reticulation. Another transect going from the groin to the knee was used to quantify the reticulation of the legs. As the front and hindlimbs share a similar pattern and colour, only the hindlimbs were used.

Finally, we also used an additional method to characterize the pattern using qualitative categories, as it has been shown that the pattern of the different *Ranitomeya* ecotypes can be successfully classified as either striped (anteroposterior lines), spotted (reticulated patterning), lined (dorsotransversal patterning band) or diffuse (uniform colour pattern on the head and random pattern on the back [35]). We use this qualitative grouping method based on the four patterning groups to correct for the high variation found within some ecotypes, and that may result in similar frogs being identified as quantitatively very different (e.g. the mimetic spotted morphs of *R. variabilis* and *R. imitator* both show high levels of variation in spot number).

To analyse the warning phenotype and compare it between individuals, each of the matrices for the 7 warning signal characteristics measured (i.e. head, body and limb pattern, head, body and limb colour + qualitative pattern) were transformed into a normalized symmetrical Euclidian distance matrix for quantitative measures, and into a symmetrical distance matrix for the qualitative variable using Dice distance. Each phenotypic matrix (*mat)* was then normalized using the following formula where *min* and *max* represent respectively the lower and higher value of the matrix.

$$\mathrm{NM}=\frac{\mathrm{mat}-\min}{\max-\min}$$

and

$$\mathrm{Matglob}=\sqrt{\begin{array}{c} (\mathrm{NMcolour}.\mathrm{hea}d^{2}+\mathrm{NMcolour}.\mathrm{bac}k^{2}+\mathrm{NMcolour}.\lim b^{2}\;\\ +\mathrm{NMpattern}.\mathrm{hea}d^{2}+\mathrm{NMpattern}.\mathrm{bac}k^{2}+\mathrm{NMpattern}.\lim b^{2}\;\\ +\mathrm{NMclassif}{.\mathrm{pattern}}^{2})\; \end{array}.}$$

These 7 normalized matrices (NM) were subsequently squared, summed and a square root transformation was applied to obtain an overall distance matrix (Matglob), effectively giving all 7 characteristics identical weight.

### 2.3. Relationship between warning signal variability and the degree of mimicry convergence

Differences in the intensity of predator selection have been documented within certain localities of the studied *Ranitomeya* [24]. With increasing predation selection for warning signal uniformity, we expect to observe a decrease in signal variation and an increase in mimetic resemblance between *Ranitomeya* species. We tested this theoretical expectation of a negative relationship between warning signal variability and mimicry resemblance. To quantify phenotypic variation of the warning signal for each of the populations, we calculated the mean pairwise phenotypic distance between individuals within each population (*Var*). To assess the degree of convergence (from perfect mimics to incomplete or ongoing mimetic convergence), we estimated whether the population of interest is phenotypically more similar (or different) to the other sympatric species than it is from allopatric populations of conspecifics. We calculated the distance using the population centroids. Dividing the phenotypic distance from its sympatric species by the mean distance from allopatric populations of conspecifics gave us the mimetic distance value . We then divided one by the sum of *Im* to transform this distance value to a measure of mimicry similarity (*ms*); this results in a value ranging from 0 to +∞, with values increasing with more perfect mimicry and values below 1 indicating no mimcry convergence is detected. This metric informs us of the amplitude of the divergence from conspecific a population underwent due to mimicry. Finally, after testing the normality of this distribution with a Shapiro-Wilk normality test (*W* ≥ 0.920, *p* ≥ 0.1966), we used a linear Pearson correlation to test for the negative relationship between warning signal variation and the degree of mimicry convergence.

### 2.4. Exploring phenotypic variability within and between ecotypes

To investigate whether phenotypic distribution is continuous or discrete between distinct ecotypes of each species, we plotted the morphospace of the quantitative measures of the warning signal using Multidimensional Scaling. Warning signals are under positive frequency dependent selection, which means that their fitness increases with their abundance. Plotting each individual phenotype enables us to visually ascertain the distribution of distinct density peaks which correspond to abundant warning signals. Theoretically, these phenotypic peaks should coincide with fitness optima separated by low fitness valleys. As it includes all localities, this visual representation (i.e. morphospace) depicts how the fitness associated with warning signals is geographically structured. The exploration of the shape (height, variability and distance between them) of the distinct peaks within the morphospace highlights the possible effect of geographical differences in natural selection in constraining, or not, the warning signals of each population. This also enables us to ascertain if the phenotypic distribution is discrete or continuous between the different ecotypes.

### 2.5. Variation within colour and/or pattern elements versus whole phenotypes

To assess whether some characteristics of the warning signal are less variable than others, possibly as a result of stronger stabilizing selection, we compared the phenotypic variability for each of the 6 quantitatively measured characteristics of the warning signal within each locality and species. The phenotypic variability was obtained by calculating the mean phenotypic distance between every combination of two individuals of the same population for a given phenotypic characteristic. We also computed the sum of the variance for each characteristic among all populations. A Kruskal-Wallis test followed by a Wilcoxon test with Bonferroni correction was performed to assess whether some phenotypic characteristics of the warning signal were more variable than others within each ecotype. Similarly, we also tested whether comparable levels of variation are observed for a given phenotypic characteristic between sympatric species. This prediction is expected if predator selection acts with the same intensity and direction on the warning signal in different sympatric *Ranitomeya* species. Finally, the comparison performed between all localities for all three species can inform on whether some colour pattern characteristics are under stronger selection, suggesting they are important for predator recognition.

All the analyses were performed in R studio [45] using the packages Patternize [42] and Rstatix [46], and plot with GGplot2 [47].

## 3. Results

### 3.1. Relationship between warning signal variability and the degree of mimicry convergence

**Figure 2.**
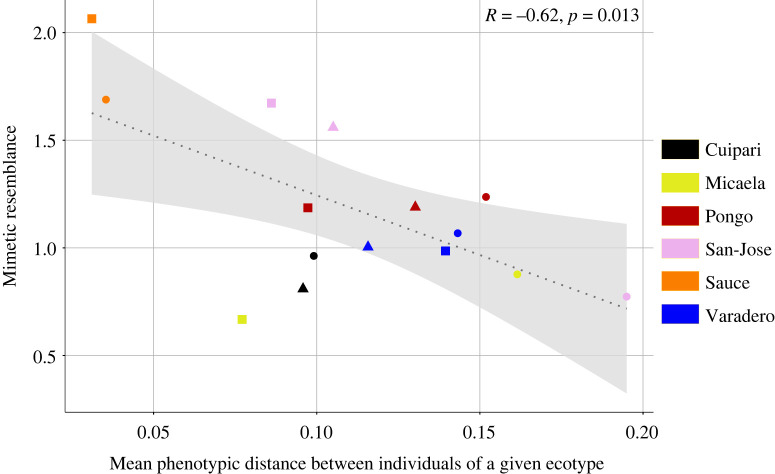
Correlation between the amount of variability within the warning signal of each ecotype and mimetic distances between each species pair in a given locality. Mean phenotypic distances between individuals of the same ecotype increases with increasing phenotypic variability within that ecotype, and mimicry resemblance increases with increasing resemblance between sympatric species. Circles: *R. fantastica* clade, squares: *R. imitator*, triangles: *R. variabilis*, dotted line: regression line, with 95% interval confidence.

We tested the relationship between local warning signal variation within species and the degree of mimetic convergence between species and found a mildly significant negative relationship (*p* = 0.01308, *d =* 13*, R* = −0.62) (figure 2). Mimicry was highly correlated with the amount of ecotypic variability in a number of sites, including Sauce and San José, where strong mimetic resemblance (from 1.56 to 2.06) coincides with low phenotypic variability (*Var* from 0.03 to 0.1), and Pongo and Varadero, where ecotypes are more variable (*Var* from 0.98 to 1.23) and little to no mimicry was observed (*ms* from 0.98 to 1.23). Interestingly, in San José this applies to only 2 of the 3 co-occurring species, with the third (*R. fantastica*) being non mimetic (*ms* = 0.77) and phenotypically very variable (*Var* = 0.19). Similarly, in Micaela, *R. fantastica* is very variable (*Var* = 0.16) and non-mimetic with *R. imitator*, which itself is much less variable (*Var* = 0.07). Finally, although both species in Cuipari have little phenotypic variation (*Var* = 0.09), the two are not mimetic at all (*ms* < 0.96).

### 3.2. Exploring phenotypic variability within and between ecotypes

**Figure 3.**
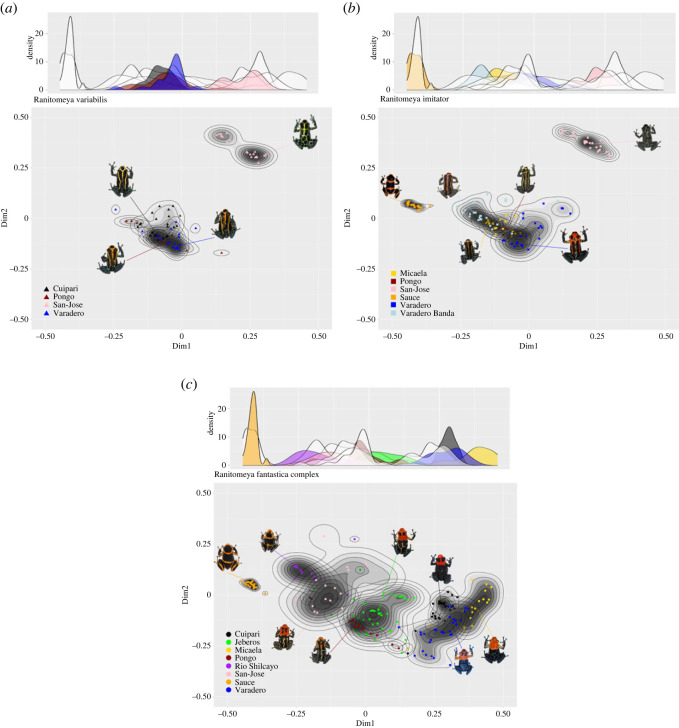
Phenotypic landscape of the warning signal using a frequency plot for (*a*) *R. variabilis* (*N* = 84), (*b*) *R. imitator* (*N* = 132) and (*c*) *R. fantastica* clade (*N* = 196), with frequency distribution of all ecotypic phenotypes above. Frequency is shown in colour for the ecotypes of the species shown. Ellipses are polygon bins of the frequency, divided into 10 levels, each of decreasing population frequency. Circle: *R. fantastica* clade, square: *R. imitator*, triangle: *R. variabilis*.

We explored the warning signal morphospace generated with the global distance matrix and observed that all three species have 1 or 2 well-differentiated peaks (figure 3). These peaks are in the same localities, for the population of Sauce and San José (respectively *R. variabilis* and *R. imitator)*, which is consistent with the mimicry relationship observed in the previous section. A binomial distribution of the phenotype is observed in San José, both for *R. variabilis* and *R. imitator*. This mostly results from a reticulation gradient, between highly reticulated to less reticulated, observed in both species. Ecotypes observed in a third site, Cuipari, also display less phenotypic variability (well-defined peaks), but phenotypic resemblance nevertheless overlaps with those of other ecotypes. In fact, with the exception of Sauce and San José, all ecotypes have gradual and overlapping clines, with some individuals resembling nearby peaks rather than their own, and/or appear to be intermediate. For *R. variabilis*, each ecotype overlaps to some degree with the others (figure 3*a*), whereas for *R. imitator* and *R. fantastica*, phenotypes overlap in a more continuous fashion (figure 3*b,c*). This is especially true for *R. fantastica*: despite clear peaks for each ecotype, there is also continuous variation along the valleys between peaks. Some individuals (approximately 6% to 29% depending on populations) harbour warning signals that are phenotypically more similar to that of other localities (alternative peak) than they are to the most abundant phenotype in their locality. In some cases, these ‘outlier’ individuals are even found within another locality's adaptive peak.

### 3.3. Variation within colour and/or pattern elements versus whole phenotypes

**Figure 4:.**
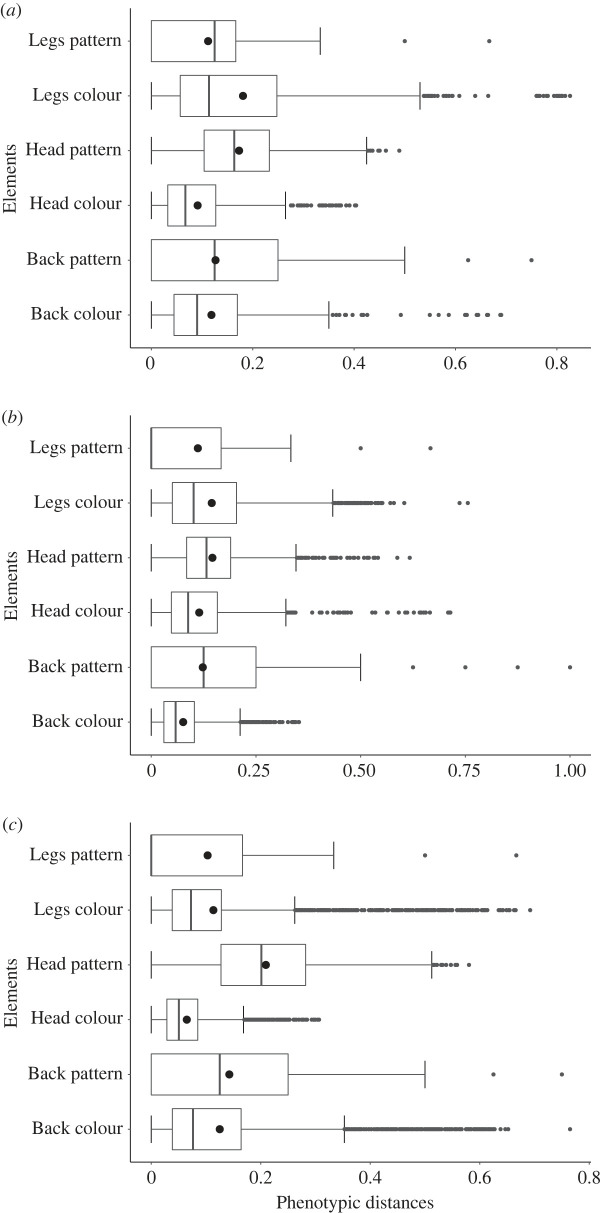
Boxplot showing the sum of the variation measured as pairwise phenotypic distance in the warning signal characteristics between every individual of (*a*) *R. variabilis*, (*b*) *R. imitator* and (*c*) the *R. fantastica* clade.

Of the 6 quantitative phenotypic characteristics measured (head, dorsal and limb colour and pattern investigated separately), all appear to display variability (variation of characteristics for a population above 12.6% of the total variation within the study system) for all ecotypes and all species (*H* ≥ 115.96, *d.f. = 5*, *p* < 0.005), as would be expected if some characteristics of the warning signal were universally under selection (figure 4). Even for those localities previously found to be less variable and more mimetic (i.e. Sauce and San José; figure 2), the least variable characteristics of the colour pattern differed somewhat between species. Nevertheless, overall the back and head colour were repeatedly found to be the least variable (*mean Var* of all ecotypes < 0.114), and therefore possibly under the strongest selection, for all ecotypes and all species. This was especially true for ecotypes with the least variation in the overall warning signal (from Sauce, San José, Cuipari). Furthermore, for all species and populations, the head pattern was found to be consistently the most variable (*mean Var* of all ecotypes = 0.188 *p* < 0.05).

## 4. Discussion

The diversification of adaptive traits strongly depends on the amount of standing variation that is present [1]. Aposematism, whereby unprofitable species advertise this fact to predators, often via bright colour patterns, is an extreme case where positive frequency-dependent selection is expected to limit the phenotypic variation present [3], even among species (i.e. mimicry). Yet, numerous aposematic species display extensive geographical divergence in warning signals throughout their range, resulting in a complex and stable spatial mosaic of colour-patterns [13,28,48]. Here, by confronting the theoretical expectation of selection on warning signals to the reality of a natural system, we show both the relationship between trait variation and mimicry, and how standing phenotypic variation may be present and facilitate warning signal diversification.

The Peruvian *Ranitomeya* system, with its extensive level of geographical diversity and mimicry relationships, enabled us to empirically demonstrate that locally an increase in mimicry resemblance between species is associated with a reduction of phenotypic variation within populations. This negative relationship is consistent with the expectation that both aposematism and Müllerian mimicry are shaped by the same positive frequency-dependent selection in nature [15]. Indeed, some localities presumably categorized by intense selection were characterized by perfect mimicry and low intra-specific variation in warning signals, whereas mimicry was absent or imperfect and warning signals were variable within species in other sites, suggesting a relaxation of predator selection and the existence of heterogeneity in the intensity of selection between localities [4]. However, notable exceptions to the negative relationship between warning signal variations and mimicry convergence were also observed, namely 1) the coexistence of two species with very distinct and fixed (i.e. non-variable) warning signals, and 2) localities where there is mimetic convergence despite high intraspecific phenotypic variations in their warning signals. Different non-exclusive explanations may exist for this.

First, developmental constraints that differ between species could hinder phenotypic convergence despite intense selection by predators. In *Ranitomeya*, an important number of patterning and pigment genes, principally related to melanophores, melanin, iridophores and guanine synthesis, are responsible for the diverse warning signals. Notable interspecific differences in the gene pathways underlying the production of the same warning signal have recently been demonstrated [49]. These differences in the pathways underlying colour pattern development between species are also supported by the recent work of Twomey *et al*. [50], which demonstrated that the colour of these frogs results from the physical interaction of nanostructure and pigments, either synthesized or acquired via dietary uptake depending on the species. The production of a novel colour or pattern with a mimetic advantage might therefore be unlikely when the difference between sympatric species is too large [5,51,52].

Moreover, the predator community may be able to memorize several warning distinct signals, enabling the coexistence of different aposematic phenotype in sympatry. The coexistence of multiple warning signals has been extensively documented in neotropical butterflies. In these taxa, multiple mimicry rings (i.e. mimetic relationship between multiple unrelated species) often coexist in sympatry, with up to a dozen species in at least five mimicry rings for Heliconiinae [53,54]. In the mimetic and aposematic Ithomiini butterfly tribe, up to 8 mimicry rings can exist within a given locality [55]. The persistence of these mimicry rings could be due to diverse spatio-temporal differences in distribution such as vertical stratification [56,57], distinct nocturnal roosting heights or habits [53], temporal separation in flight activity [58], etc. The different mimicry rings in these butterflies display very different signals and any intermediate forms are selected against [59,60]. In *Ranitomeya*, selection against intermediate forms is consistent with the existence of co-occurring but non-mimetic species, where each warning signal is visually very different (See Cuipari for exemple, figure 1, figure 2).

On the other hand, large within population signal diversity despite mimicry could result from the migration of new warning signal alleles from other populations. This connectivity between ecotypes has been proposed to maintain phenotypic variation even when the signal is under positive frequency-dependent selection [61,62] (figure 2). The effect of gene flow has been documented in a number of study systems, including in the hybrid zones of *Heliconius* [63,64], where large numbers of recombinant phenotypes are observed within long but narrow regions of contact between races. Similar situations have been documented in poison dart frogs, such as in *Oophaga anchicayensis* and *Oophaga lehmanii* [65], and between the mimetic ecotypes of *R. imitator* [35], where hybrid zones are characterized by large amounts of warning signal diversity.

Furthermore, predator generalization at the community level could lead to species displaying accurate mimicry despite variability in the warning signal. Generalization can be observed when predators learn to avoid an unpalatable phenotype and as a result will also avoid other more or less similar forms, thus enabling some level of variation to be maintained [33]. Indeed, a limited number of key aspects of the warning signal may be used for recognition by predators. While these traits are expected to be less variable, the rest of the colour-pattern might be under relaxed selection and could therefore be more variable. Indeed, our dissection of the warning signal revealed that the head colour, and to a lesser extent the back colour, were the least variable traits, not only within populations characterized by a more variable warning signal (e.g. Cuipari, Pongo and Varadero), but for all populations and species in general (figure 4). Patterning, however, was consistently the most variable aspect of the signal.

These differences inherent to different components of the warning signal are consistent with other analytical and experimental studies: long-wavelength colours (i.e. red, orange and yellow) that characterize the head and dorsum of *Ranitomeya* are thought to provide the most efficient and contrasting signal in the forest environment, making coloration a key aspect of an effective warning signal and under strong stabilizing selection [66]. Moreover, bird predators have been shown to preferentially avoid colour rather than other phenotypic elements in aposematic prey [32] enabling those other characteristics to be more variable. Finally, the patterns created by the alternating vivid colour and black background, while surely improving detection and learning by predators, are also easily generalized by avian predators [67]. As such, different patterns, such as varying degrees of reticulations, are likely not discriminated by predators, enabling for variations in the local warning signal. Moreover, these patterning elements are likely generated by reaction–diffusion mechanisms which result in unique patterns for each individual [43] and even continued change during an individual's lifetime [68]. The low diversity of colours compared to patterning elements could also be explained by sexual selection acting to stabilize this trait. While little is known about mate choice in *Ranitomeya*, a major role of colour in communication has been documented in the strawberry poison frog (*Oophaga pumilio*); in this species, females prefer brighter male dorsal coloration with apparently no preference for other warning signal characteristics [69,70].

Finally, our analyses of the morphospaces revealed that the phenotypic distribution of each ecotype is not as discrete or as constrained as initially expected (figure 3). While a number of discrete mimetic ecotypes are observed (e.g. the green reticulated *R. variabilis* and *R. imitator*, the orange banded *R. imitator* and *R. summersi*), variations within some localities overlap with those of other localities, such that differentiation between ecotypes is blurred. This is especially apparent in the *R. fantastica* clade where numerous localities have a few individuals that are distant from the local warning signal optimum (i.e. the most abundant one). While the source of this within-locality phenotypic variation cannot currently be ascertained (i.e. migration, relaxation of predation, warning signal generalization, etc.), the standing variation present locally may fuel the diversification of warning signals. Population with warning signal variation can be the source of the colonization of a new habitat with a naive predator community and therefore generate the birth of a new ecotype [24].

In conclusion, assessing how variations in the warning signals of *Ranitomeya* frogs are structured at the scale of individuals, populations, geography and communities between unrelated species has enabled us to challenge and confirm, to some extent, theoretical expectations of natural selection and diversification on this iconic trait. As expected, we show a negative relationship between signal variability and mimetic convergence, demonstrating experimentally that both phenomena are the result of positive frequency selection. But contrary to general expectations, we show that warning signals are much more variable than expected, and that variability between population seems continuous, suggesting the maintaining of this variability among the metapopulation. This standing genetic variation is most likely the key to warning signal diversification: founding effects might strongly contribute to colour pattern diversification.

## Data Availability

The data are available from the Dryad Digital Repository: https://doi.org/10.5061/dryad.q2bvq83qq [71].
